# Th17 Cells Exhibit Antitumor Effects in MDS Possibly through Augmenting Functions of CD8+ T Cells

**DOI:** 10.1155/2016/9404705

**Published:** 2016-09-19

**Authors:** Jing Li, Lanzhu Yue, Huaquan Wang, Chunyan Liu, Hui Liu, Jinglian Tao, Weiwei Qi, Yihao Wang, Wei Zhang, Rong Fu, Zonghong Shao

**Affiliations:** Department of Hematology, The General Hospital of Tianjin Medical University, Tianjin, China

## Abstract

Th17 cells are a newly found subset of distinct CD4+ Th effector cells' family and are found to play an important role in cancers. Myelodysplastic syndromes (MDS) are a common malignant hematological disease. Here, we showed that both the percentage and the function of Th17 cells were elevated in low-risk MDS while being decreased in high-risk MDS. Levels of upstream molecules of Th17 cells, IL-6 and IL-23, were higher in low-risk MDS but lower in high-risk MDS patients. The abnormal percentage of Th17 cells was closely related to clinical parameters including karyotype, morphologic blast percentage of bone marrow, peripheral absolute neutrophil count, and hemoglobin concentration. Furthermore, expression rates of perforin and granzyme B in BM CD3+CD8+ cells (cytotoxic T lymphocyte, CTL) positively correlated with levels of IL-17 but negatively correlated with BM blast percentage and could be significantly increased after stimulation with human recombinant IL-17 (rhIL-17). Our results suggested that Th17 cells might play an antitumor effect in the pathogenesis of MDS through IL-17/CTL pathway.

## 1. Introduction

Myelodysplastic syndromes (MDS) are a diverse group of clonal hematopoietic malignancies characterized by ineffective hematopoiesis, progressive bone marrow failure, cytogenetic and molecular abnormalities, and unpredictable risk of further deteriorating into acute myeloid leukemia (AML) [1]. Pathogenesis of MDS is complicated and sustained by a burden of recurrent molecular, cytogenetic, and epigenetic defects. Numerous clinical and experimental data suggest the involvement of T lymphocytes in the pathogenesis of MDS; however the actual weight exerted by T cells in this scenario is yet to be conclusively dissected [2]. Previous studies from our group and others found that the number of Th1 cells was decreased with Th2 cells increasing relatively resulting in an imbalance of Th1/Th2, which was proved to be correlated with condition of MDS patients [3]. What is more, the frequency of Treg cells was elevated with hyperfunction and was obviously correlated with the disease condition of MDS patients [4–6]. Patients with high-risk MDS also had higher frequency of T regulatory cells than normal [7], which suggested that progression of MDS was facilitated by immune suppression and tumor immune deficiency. Lately even Th17 cells have been advocated in the pathogenesis of MDS for the first time [8].

Th17 cells, named by their signature cytokine IL-17, are a distinct subset of CD4+ Th effector cells with RAR-related orphan receptor *γ*t (ROR*γ*t) and signal transducers and activators of transcription-3 (STAT-3) as the transcription factor to direct their differentiation. Many researches have clarified the importance of Th17 cells in inducing inflammation and autoimmune tissue injury to defend against microbial infections. However, the role of Th17 cells acting in the pathogenesis of malignant diseases remains equivocal as the available data showed a contribution of Th17 cells to either protective antitumor immunity or promoting tumor growth by IL-17 and IL-23 [9, 10]. Although more and more results have supported the antitumor effects of Th17 cells by eradicating tumor cells directly or recruiting other tumor-specific immune cells and/or by promoting their antitumor immune responses [11], most data are derived from murine models; the role and pathway of human Th17 cells in antitumor immunity are still incompletely defined.

Our present study seeks to illuminate roles of Th17 cells in the pathogenesis of MDS patients in different risk groups through measuring the frequency of Th17 cells, detecting levels of related cytokines in patients with different risks of MDS, analyzing the correlation between Th17 cells percentage and hematological parameters of MDS patients, and testing levels of cytotoxic molecules expressed in CTL before and after human recombinant IL-17 (rhIL-17) stimulation, ultimately to explore the role of Th17 cells in the tumorigenesis of MDS.

## 2. Materials and Methods

### 2.1. Study Populations

A total of 42 patients (18 females and 24 males; range from 21 to 84; median age 58 years) with MDS were enrolled in this study. Patients were treatment-naive when sampling. All were inpatients of the Hematology Department of General Hospital Tianjin Medical University, China, from March 2015 to February 2016. The diagnosis of MDS was based on WHO classification [12], including one refractory thrombocytopenia (RT), three refractory anemia (RA), six refractory anemia with ringed sideroblasts (RARS), three 5q- syndrome, eight refractory cytopenia with multilineage dysplasia (RCMD), seven refractory anemia with excess blast I (RAEB I), and fourteen RAEB II patients. Patients were excluded if they had autoimmune diseases, infection, or other malignant diseases and were further divided into two groups based on International Prognostic Scoring System (IPSS) score [13]: low-risk MDS (L-MDS, IPSS ≤ 1.0, *n* = 22) and high-risk MDS (H-MDS, IPSS > 1.0, *n* = 20). Characteristics of patients are shown in Table 1. Eighteen healthy controls (14 females and 9 males) with a median age 57 years (range 19 to 71) were enrolled in this study, including 8 males and 11 females. The study was approved by the Ethics Committee of the Tianjin Medical University, China. Informed written consent was obtained from all patients or their parents in accordance with the Declaration of Helsinki.

### 2.2. Preparation of Mononuclear Cells and Plasma

Fresh peripheral whole blood (PB) and bone marrow (BM) were collected. Serum was obtained by centrifugation and stored at −80°C for cytokine analysis. Peripheral blood mononuclear cells (PBMNCs) and bone marrow mononuclear cells (BMMNCs) were isolated by gradient centrifugation (400 g, 20 minutes) using Ficoll-Paque (Solarbio, Shanghai, China) for flow cytometric analysis and RNA isolation.

### 2.3. Intracellular Staining and Flow Cytometric Analysis to Detect the Percentage of Th17 Cells

In order to stain the intracellular cytokine and analyze surface phenotype by flow cytometry (FCM), 2 × 10^6^ PBMNCs or BMMNCs were resuspended in 2 mL Roswell Park Memorial Institute (RPMI) 1640 medium with 10% fetal bovine serum (FBS, Solarbio, Shanghai, China) and incubated for 5 h at 37°C, 5% CO_2_ in the presence of 25 ng/mL of phorbol myristate acetate (PMA), 1 *μ*g/mL of ionomycin, and 10 *μ*g/mL of Brefeldin A (all from Beyotime Biotechnology, Shanghai, China). PMA and ionomycin are pharmacological T cell-activating agents that simulate signals generated by the T cell receptor (TCR) complex and stimulate T cells of any antigen specificity. Brefeldin A (BFA) was used as an intracellular protein transport inhibitor, thereby leading to an accumulation of cytokines in the cells. After incubation, cells were stained with anti-CD3-APC and anti-CD4-FITC monoclonal antibodies at room temperature in dark for 20 min. And after fixation and permeabilization, the cells were next stained with anti-IL-17-PE monoclonal antibody as well as an isotype control to enable correct compensation and confirm antibody specificity. All antibodies and solutions were purchased from BD Biosciences (Franklin Lakes, NJ, USA). Th17 cells are identified as CD3+CD4+IL-17+. Finally, a minimum of 50,000 cells were acquired on a FACS-Calibur flow cytometer (BD Biosciences) and analyzed by Cell Quest software version 3.1.

### 2.4. Detection of IL-17, IL-6, and IL-23 by Enzyme-Linked Immunosorbent Assay (ELISA)

To determine the concentration of IL-17, IL-6, and IL-23, the serum was analyzed by ELISA (R&D Systems, Minneapolis, MN, USA) following the manufacturer's suggested protocols.

### 2.5. Determination of the Expression of IL-17, ROR*γ*t, and STAT-3 mRNA by qRT-PCR

Total RNA in PBMNCs and BMMNCs was extracted using TRIzol reagent (Invitrogen) and reversed transcribed into cDNA using PrimeScript RT reagent kit (Takara Bio Inc., Dalian, China). The sequences of primers specific for the target genes are shown in Table 2. Quantitative reverse transcription polymerase chain reaction (qRT-PCR) was performed for IL-17, ROR*γ*t, and STAT-3 on an BIO-RAD iQ5 Real-Time System (BIO-RAD, USA) using SYBR Premix Ex Taq II (Takara Bio) as a double-strand DNA-specific binding dye. Human GAPDH gene was used as an endogenous control for sample normalization. Primer sequences (Table 2) were designed and generated by Gene-wiz Bio Inc., Suzhou, China. After normalization of the data according to the expression of GAPDH mRNA, the levels of target mRNA were calculated using the 2-ΔΔCt method ((Ct, target gene-Ct, GAPDH) sample - (Ct, target gene-Ct, GAPDH) control).

### 2.6. Detection of Perforin (Per) and Granzyme B (GB) in CTL before and after rhIL-17 Stimulation

Expression rate of cytoplasmic Per and GB of CTL in BM was analyzed by FCM before and after rhIL-17 stimulation. Experiment was carried out as follows: 4 × 10^6^ BMMNCs were resuspended in 24-well plates with 4 mL RPMI 1640 medium containing 10% FBS and incubated for 24 h at 37°C, 5% CO_2_ in the presence of 50 ng/mL rhIL-17. After the specified incubation period, cells were collected and stained with anti-CD3-PerCP and anti-CD8-FITC monoclonal antibody at room temperature in dark for 20 min. And after fixation and permeabilization, cells were next stained with anti-Per-APC and anti-GB-PE monoclonal antibodies as well as their isotype control to enable correct compensation and confirm antibody specificity. All antibodies and solutions were purchased from BD Biosciences (Franklin Lakes, NJ, USA). Finally, a minimum of 20,000 cells were acquired on a FACS-Calibur flow cytometer (BD Biosciences) and analyzed by Cell Quest software version 3.1.

### 2.7. Statistical Analysis

All analyses were performed using SPSS 21.0 software (SPSS Science). Data were presented as mean ± standard deviation (SD). *t*-test and one-way ANOVA test were applied to analyze normality data. Spearman's rank correlation test was used for correlated data. A value of *P* < 0.05 was considered statistically significant.

## 3. Results

### 3.1. Th17 Cells Are Elevated in L-MDS Patients While Being Decreased in H-MDS Patients

**Figure 1:**
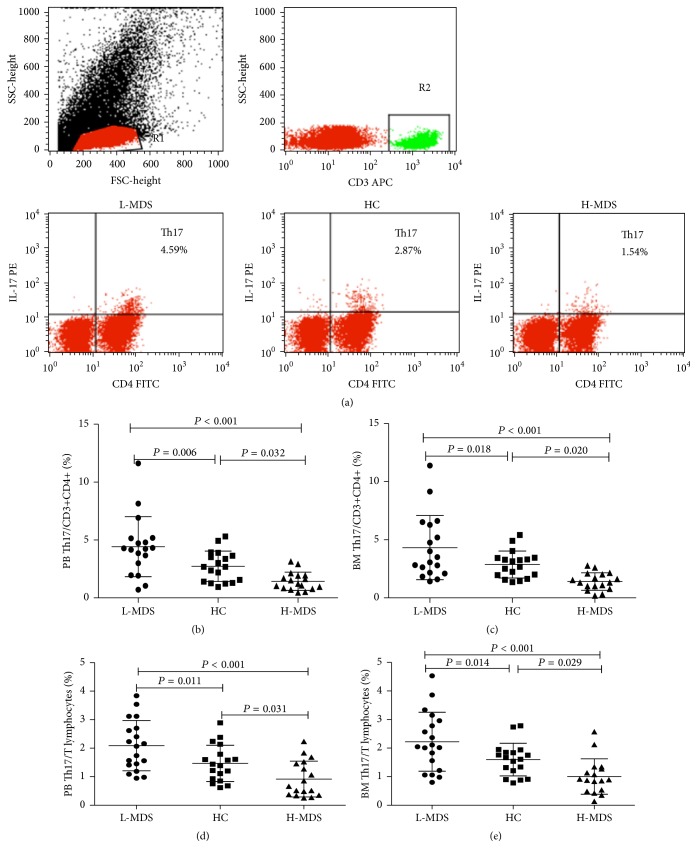
Th17 cells expand differentially in L-MDS and H-MDS patients. Lymphocytes were gated by flow cytometry. Representative FACS dot plots of Th17 (CD4+IL-17+) cells from L-MDS, HC, and H-MDS patients (a). Percentages of PB (b) and BM (c) Th17 cells in CD4+ T cells were shown. Percentages of Th17 cells in total T lymphocytes in PB and BM were also shown in (d) and (e), respectively.

Lymphocytes were gated by flow cytometry and representative FACS dot plots of Th17 (CD4+IL-17+) cells from L-MDS patients, healthy controls (HC), and H-MDS patients were shown in Figure 1(a). In order to identify potential mechanisms of Th17 cells in the pathogenesis of MDS, we initially assessed the percentage of both Th17 cells/CD3+CD4+ cells and Th17 cells/CD3+ cells (T lymphocytes) in PB and BM of patients with different risks of MDS (Figures 1(b)–1(e)). Compared with HC, PB Th17 cells of L-MDS patients displayed significantly higher frequency (4.42 ± 2.59%) compared to those of HC (2.73 ± 1.32%, *P* < 0.01) and H-MDS patients (1.42 ± 0.79%, *P* < 0.01), and there was also a significant difference between the latter two groups (*P* < 0.05, Figure 1(b)). Analogous findings as regards the percentage were observed in the BM of L-MDS (4.32 ± 2.76%, *P* < 0.01) and H-MDS (1.37 ± 0.84%, *P* < 0.05, Figure 1(c)) patients comparing with HC (2.93 ± 1.21%). Consistent tendency was shown in the percentage of PB as well as BM Th17/T lymphocytes(Figures 1(d)-1(e)).

### 3.2. The Percentage of Th17 Cells Negatively Correlates with BM Blast Percentage and Positively Correlates with Peripheral Blood Cell Counts

**Figure 2:**
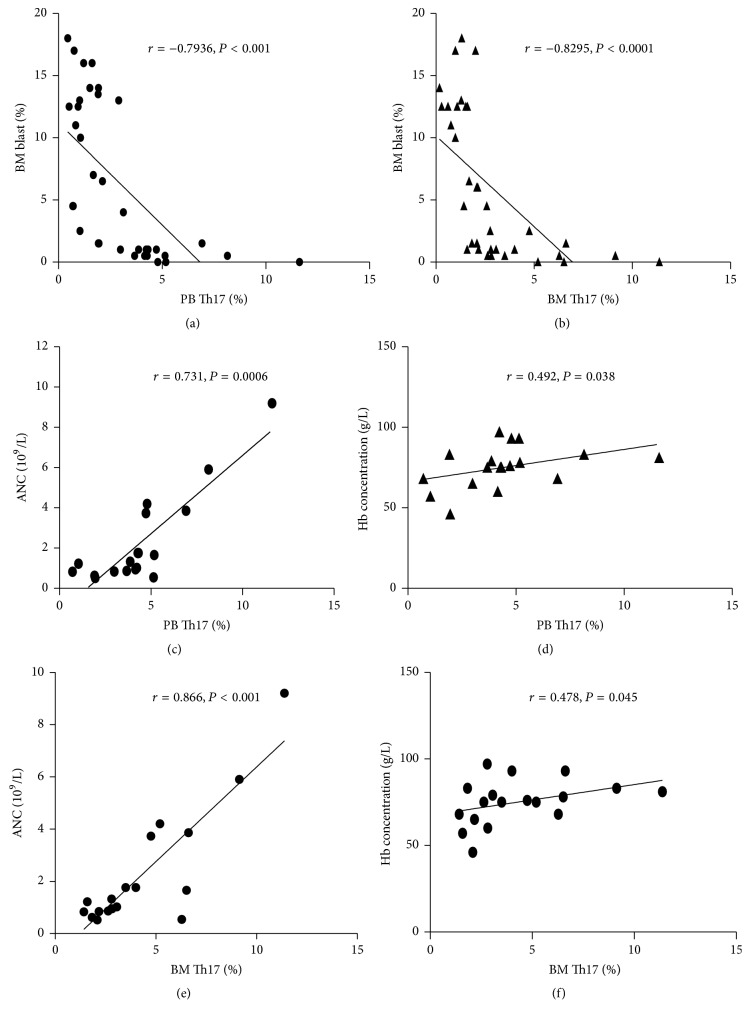
Th17 percentages negatively correlate with BM blast ratios and positively correlate with peripheral cell counts. Correlation analyses were performed between BM blast ratios and PB (a) or BM (b) Th17 percentages. Relationships between peripheral blood cell counts and PB (c-d) or BM (e-f) Th17 percentages were also analyzed.

To further figure out the role of Th17 cells, we checked the relationship between the Th17 cells/CD3+CD4+ cells percentage (the percentage of Th17 cells) and bone marrow blast percentages as well as peripheral cell counts. Our results showed that the percentage of both PB and BM Th17 cells negatively correlated with BM blast percentage (BM blast%) (Figures 2(a)-2(b)), suggesting that Th17 cells might be playing a protective role against the malignant clone in MDS.

We further analyzed the correlation between Th17 percentage and peripheral cell counts in L-MDS patients. Interestingly, data displayed a positive relation of the percentage of PB Th17 cells with absolute neutrophil count (ANC) (*r* = 0.875, *P* = 0.0001, Figure 2(c)) and hemoglobin (Hb) concentration (*r* = 0.492, *P* = 0.038, Figure 2(d)). Similar positive relations were found between the percentage of BM Th17 cells and ANC (*r* = 0.856, *P* = 0.0006, Figure 2(e)) as well as Hb concentration (*r* = 0.478, *P* = 0.045, Figure 2(f)). This indicated that Th17 cells might specifically kill malignant clones while sparing normal cell populations.

### 3.3. Th17 Cells Contribute to Favorable Cytogenetic Phenotypes

**Figure 3:**
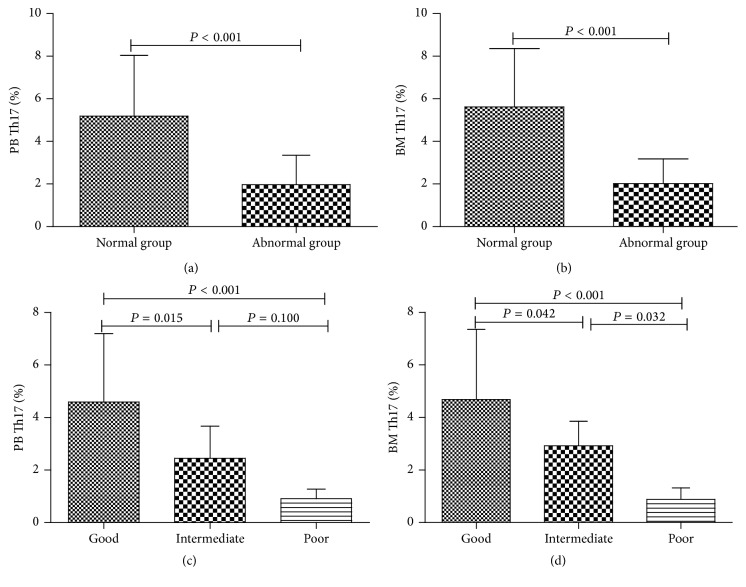
Th17 cell percentages differ among different karyotype groups. Differences of Th17 percentages between normal and abnormal karyotype groups were shown (a-b). PB and BM Th17 percentages in MDS patients with good, intermediate, and poor karyotypes were shown in (c) and (d).

We next looked at the correlation between Th17 cells and karyotypes. According to karyotype analysis, patients with MDS were first divided into normal karyotype (*n* = 12) and abnormal karyotype group (*n* = 23) and significant differences in the percentage of both PB and BM Th17 cells were seen between the two groups (Figures 3(a)-3(b)). Then patients were further categorized as good karyotype group (*n* = 17, including normal, −Y, del(5q), and del(20q)), poor karyotype group (*n* = 10, including complex, chromosome 7 abnormality), and intermediate karyotype group (*n* = 8, including all other abnormalities); percentage of PB and BM Th17 cells was significantly higher in good karyotype group and obviously lower in poor group compared with intermediate group (*P* < 0.05, Figures 3(c)-3(d)).

### 3.4. Differential Percentages of Th17 Cells in MDS Might Be Driven by Different Levels of Upstream Cytokines

**Figure 4:**
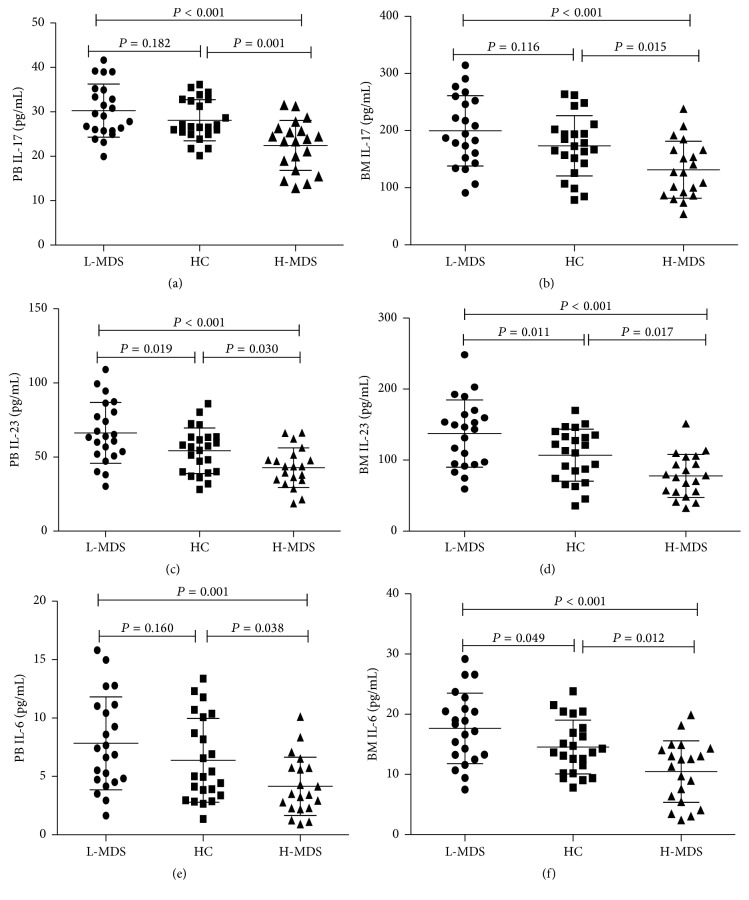
Differential expression of upstream cytokines might be driving the changes in Th17 numbers. PB and BM levels of IL-17 (a-b), IL-23 (c-d), and IL-6 (e-f) in L-MDS, HC, and H-MDS groups were measured by ELISA.

To find out possible mechanisms involved in the regulation of Th17 percentages, we measured several cytokines including IL-17, IL-6, and IL-23 levels in PB and BM serum of patients with MDS and HC by ELISA. Results showed that levels of PB IL-17 in patients with H-MDS (22.44 ± 5.64 pg/mL) were markedly lower than those in patients with L-MDS (30.29 ± 5.97 pg/mL, *P* < 0.001) and HC group (28.11 ± 4.64 pg/mL, *P* = 0.01) (Figure 4(a)); levels of BM IL-17 in H-MDS group (131.67 ± 49.71 pg/mL) were also significantly lower than those in patients with L-MDS (199.71 ± 61.49 pg/mL, *P* < 0.001) and HC group (173.59 ± 52.70 pg/mL, *P* = 0.015) (Figure 4(b)). As for levels of IL-6 and IL-23 in PB and BM, similar tendencies were seen among L-MDS, HC, and H-MDS groups (Figures 4(c)–4(f)).

### 3.5. Critical Transcription Factors of Th17 Cells Are Differentially Expressed in MDS

**Figure 5:**
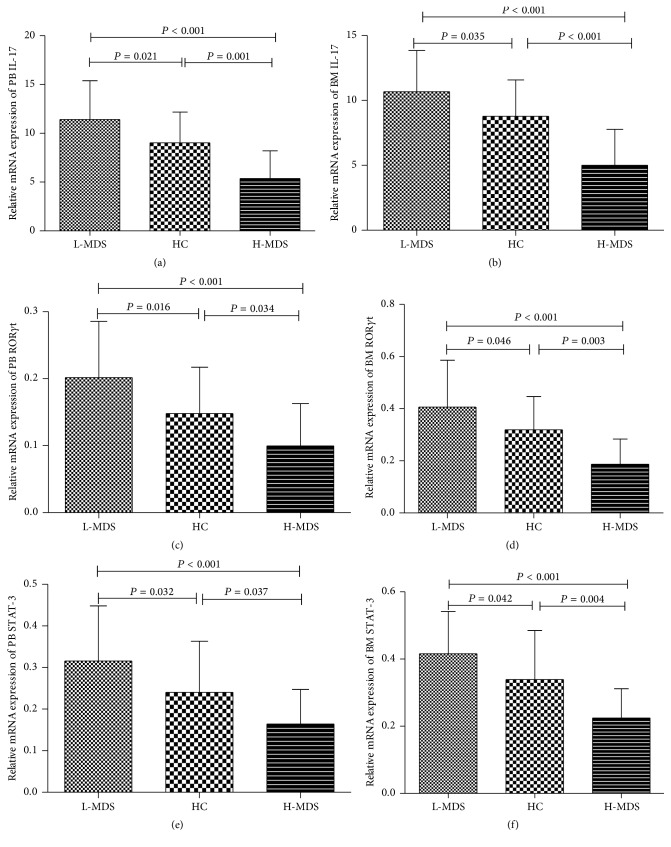
Critical transcription factors of Th17 cells are differentially expressed in MDS patients and controls. mRNA levels of IL-17 (a-b), ROR*γ*t (c-d), and STAT-3 (e-f) in PB and BM from L-MDS, HC, and H-MDS groups were determined by qRT-PCR.

Consistent with levels of IL-17, levels of IL-17 mRNA in L-MDS, HC, and H-MDS group were 11.81 ± 4.77, 7.46 ± 3.05, and 4.72 ± 2.91 in PB and 10.66 ± 3.18, 8.78 ± 2.84, and 5.00 ± 2.76 in BM; *P* values were all <0.05 between any two groups (Figures 5(a)-5(b)). Relative expression levels of PB and BM ROR*γ*t and STAT-3 mRNA were significantly decreased in H-MDS group compared to L-MDS and HC groups, consistent with the levels of IL-17 mRNA (Figures 5(c)–5(f)).

### 3.6. Th17 Cells Possibly Function through Increasing Per and GB Production by CTL

**Figure 6:**
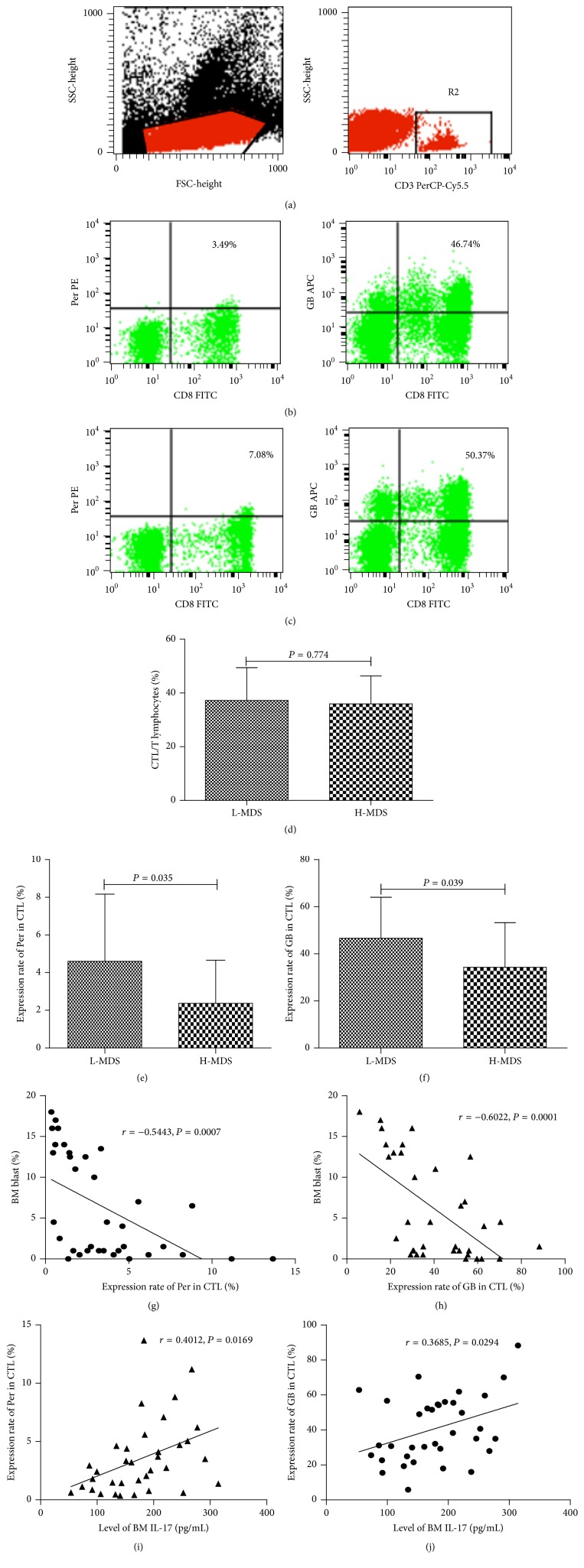
Correlations of Per as well as GB expressed in CTL and IL-17. Lymphocytes were gated by flow cytometry (a). Representative FACS dot plots of Per+ and GB+ CTL cells before rhIL-17 stimulation (b) and after rhIL-17 stimulation (c) were shown. The CTL percentages were shown in (d); also the expression rates of Per and GB were shown in (e-f). Correlations between Per, GB expression, and bone marrow blast ratios (g-h) as well as levels of IL-17 (i-j) were analyzed.

Lymphocytes were gated by flow cytometry (Figure 6(a)) and representative FACS dot plots of Per+ as well as GB+ expressed in CTL cells were shown in Figures 6(b)-6(c). Percentage of CTL in L-MDS and H-MDS were compared and there was no significant difference between the two groups (Figure 6(d)). We further tried to explore the pathway through which Th17 cells exerted their effects and found that comparing with H-MDS group, the expression of Per, GB in CTL of patients with L-MDS was notably higher (Figures 6(e)-6(f)); correlation analysis showed that the expression of Per and GB in CTL negatively correlated with BM blast% (Figures 6(g)-6(h)) and positively correlated with levels of BM IL-17 (Figures 6(i)-6(j)).

**Figure 7:**
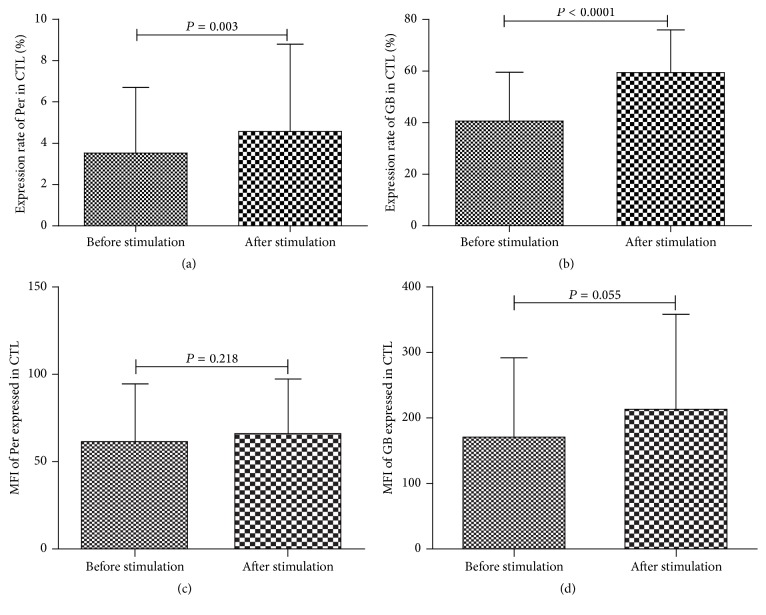
Th17 cells exert antitumor effects possibly through increasing Per and GB production by CTL. Expressions of Per and GB in CTLs before and after rhIL-17 stimulation were shown in (a) and (b). MFI of Per and GB expressed in CTL before and after rhIL-17 stimulation were shown in (c) and (d).

Therefore, we then stimulated BMMNCs of MDS patients with rhIL-17 and assessed changes in Per and GB expressions of CTL. Paired *t*-test was used for data analysis; results showed that the percentage of CTL expressing Per was significantly increased after rhIL-17 stimulation (*P* = 0.003); as for the expression rate of GB in CTL of MDS patients, a marked increase after stimulation was also seen (*P* < 0.0001, Figures 7(a)-7(b)). To further determine Per and GB expression level in each cell, mean fluorescence intensity (MFI) of Per and GB expressed in CTL was assessed. Although a slight elevation of GB expression was observed, no significant increase was found regarding Per and GB expression after rhIL-17 stimulation (*P* > 0.05, Figures 7(c)-7(d)). These findings indicate that Th17 cells possibly function through improving Per and GB production mainly by increasing the number of CTLs that express Per and GB other than on a per-cell basis.

## 4. Discussion

MDS are malignant clonal disease of hematopoietic system which have a high risk of further deteriorating and progressing to AML. Pathogenesis of MDS could be concluded as age-induced genetic, epigenetic, and immune-mediated changes in haemopoietic stem cells (HSC) which lead to oligoclonal expansion of myelodysplastic stem cells, with defective differentiation contributing to microenvironmental changes and immune deregulation, characterized by increased apoptosis of erythroid and myeloid progenitors [1].

Nowadays, impairment of the immune surveillance has been highlighted in the pathogenesis and evolution of MDS through studies on Treg cells [4, 14, 15], natural killer (NK) cells [16], helper T cells including Th1 and Th2, and the ratio of them [3, 17, 18]. In recent years, as a new subtype of helper T cells characterized by the specific cytokines and transcription, the potential role of Th17 cells in the MDS pathogenesis has been intensively addressed. Noteworthy, roles of this cell subset remained to be controversial, overrepresented in L-MDS compared with H-MDS [8, 19]; in contrast, Bouchliou et al. believed that Th17 cells were significantly decreased and hypofunctional in L-MDS patients when compared with that in H-MDS patients [20].

As we all know, Th17 cells are a newly found subset of distinct CD4+ Th effector cells' family named by their signature cytokine IL-17 and are found to play an important role in tumor disease [8–10]. Our present study found that compared with HC, circulating Th17 cells in both PB and BM was significantly elevated in L-MDS group and decreased in H-MDS group, in accordance with researches of Hamdi et al. [18] and Shao et al. [19]. Meanwhile, correlation analysis showed that the percentage of Th17 cells in normal karyotype group was higher than that in abnormal karyotype group markedly, and the poorer the karyotype was, the lower the percentage of Th17 cells was. What is more, while a positive relation was seen between the percentage of PB and BM Th17 cells of L-MDS patients and their ANC and Hb concentration, negative relations between the percentage of BM and PB Th17 cells in MDS patients and morphologic blast count in bone marrow smear were displayed; all of above led us to believe that Th17 cells mainly played a protective role in the pathogenesis of MDS and could relieve the inhibition of malignant clone on normal hematopoiesis while selectively killing malignant clone, maintaining the relative advantage of normal hematopoiesis and assisting in sustaining the genetic stability of MDS patients.

As the transcription factor of Th17 cells, ROR*γ*t had been identified as the master regulator of polarization of helper T cells toward the Th17 pathway. It could induce transcription of the genes encoding IL-17 and the related cytokine IL-17F in naive CD4+ T helper cells and was required for their expression in response to IL-6 and TGF-*β*, the cytokines known to induce IL-17 [21, 22]. Recently, STAT-3, a major signal transducer for IL-6 and IL-23, was recognized as a pivotal transcription factor in directing and regulating Th17 cells development [23–26], retroviral expression of a hyperactive STAT-3 enhanced Th17 cell differentiation, while STAT-3 deficiency damaged Th17 cell differentiation through weakened ROR*γ*t expression [24, 27]. Here, both of the PB and BM mRNA expression levels of IL-17, the specific function molecule of Th17 cells, were higher in L-MDS group than in H-MDS group, in accordance with the concentration of IL-17 in serum. Also the PB and BM mRNA expression levels of ROR*γ*t and STAT-3, transcriptional factors of Th17 cells, were markedly higher in L-MDS patients and lower in H-MDS patients compared to healthy persons, implying that not only the percentage but also the functional and activation state of Th17 cells displayed an expansion in L-MDS and a sag in H-MDS.

IL-6, which has been originally identified as a B-cell differentiation factor, is a multifunctional cytokine that regulates the immune response, haematopoiesis, the acute phase response, and inflammation [28].* In vitro* studies in mice have shown that the costimulation of IL-6 and TGF-*β* is essential for the differentiation of Th17 cells from naive CD4+ T cells [29]. IL-23, mainly produced by activated M1 macrophages, was a proinflammatory cytokine composing the IL-23p19 and IL-12/23p40 subunits and played a crucial role in upregulating the expression of IL-17 through regulating Th17 cell polarization by inducing the phosphorylation of STAT3, expanding and stabilizing Th17 cells [30]. Thus, as the two major upstream molecules, IL-23 and IL-6 played an important role in the differentiation and phenotype stabilization of Th17 cells. Despite reasons for changes in the percentage as well as the functional and activation state of Th17 cells remained unknown, data of our study indicated a notable rise in both PB and BM serum concentrations of IL-23 and IL-6 in L-MDS patients compared with that of H-MDS patients. Hence, we inferred that the differences in the percentage as well as the functional and activation state of Th17 cells between L-MDS and H-MDS patients might be due to the different concentrations of IL-6 and IL-23.

IL-17 was the signature cytokine of Th17; researches showed that IL-17 could enhance the cytotoxic effects of NK cells against tumor cells by augmenting the expression of cytotoxic molecules including tumor necrosis factor-*α* (TNF-*α*), interferon-*γ* (IFN-*γ*), Perforin, and Granzyme B [31]. In addition, transfection of IL-17 in immunocompetent mice but not in nude mice inhibited the hematopoietic tumor growth as a result of increased tumor-specific cytolytic T cells [32]. Enhanced tumor growth and lung metastases in IL-17-deficient mice were associated with the decreased IFN-*γ*+ natural killer cells and tumor-specific IFN-*γ*+ T cells in tumor-draining lymph nodes and tumors [33]. As two major cytotoxic molecules, Per can form pores in target cell membranes, while granzymes, as serine proteases, enter the cytoplasm of the target cells, altering their function and/or activating cell death [34]. Here, we found that both of the PB and BM mRNA expression levels of IL-17 were higher in L-MDS group than in H-MDS group, in accordance with the concentration of IL-17 in serum. What is more, expression rate of Per and GB in CTL of BM positively correlated with levels of IL-17 but negatively correlated with BM blast%, and the expression rate of Per and GB in CTL was significantly increased after stimulation with rhIL-17* in vitro*. Therefore, we believed that Th17 cells might play antitumor effects through IL-17/CTL pathway.

In conclusion, our study supported the fact that Th17 cells mainly played antitumor effects in the pathogenesis of MDS; meanwhile, they relieved the inhibition of malignant clone on normal hematopoiesis while selectively killing malignant clone, maintaining the relative advantage of normal hematopoiesis. As upstream molecules of Th17 cells, abnormal levels of IL-6 and IL-23 may be the the chief culprit that led to the abnormalities in both percentage and function of Th17 cells. Through the rhIL-17 stimulation experiment* in vitro*, we believed that Th17 cells might play antitumor effects through IL-17/CTL pathway. However, it will be necessary to further explore other pathways Th17 cells targeted* in vivo* and* in vitro* and provide more opportunities and possibilities for a better clinical therapy of MDS.

## Figures and Tables

**Table 1:** Patient characteristics^a^.

Case	Age	Sex	Diagnosis	Cytogenetics	IPSS	Blast (%)
1	61	Male	RUMD	Normal	0.5	0.5
2	59	Male	RUMD	Normal	0.5	0
3	54	Female	5q- syndrome	5q-	0.5	1
4	56	Female	RAEB I	+8,20q-	1.5	8
5	64	Male	RA	20q-	0.5	1.5
6	84	Male	RAEB II	Normal	2.0	16
7	38	Male	RAEB II	−9,−11,+8	3.0	17
8	21	Male	RUMD	Normal	0.5	2.5
9	50	Male	RAEB II	+8,20q-	2.5	14
10	27	Male	RT	+8	0	1
11	50	Female	RAEB II	+8,20q-	2.0	17
12	50	Male	RARS	+8	0	0.5
13	58	Female	RAEB II	5q-,7q-,20q-	3	18
14	61	Male	5q- syndrome	5q-	0.5	1
15	62	Female	RARS	Normal	0	1.5
16	58	Female	RAEB II	7q-,−12,−13	3.0	12.5
17	37	Male	RAEB I	+8,−Y	1.0	7
18	30	Male	RUMD	+8	0.5	1
19	69	Female	RAEBI	+8,−12	1.0	6.5
20	68	Male	RAEB II	7q-	2.0	10
21	33	Male	RARS	Normal	0	0.5
22	38	Male	RAEB II	+8,20q-	2.5	12.5
23	79	Female	RAEB II	Normal	2.0	12.5
24	58	Male	RAEB I	Normal	1.0	8.5
25	62	Male	RUMD	5q-,20q-	1.0	1
26	53	Female	RAEB II	−14	2.5	11
27	29	Female	RAEB II	Normal	2.0	16
28	70	Female	RAEB II	−7,−14	3.0	13
29	49	Female	RARS	Normal	0	0.5
30	53	Male	RAEB I	−7,−13	2.0	6
31	59	Female	RAEB I	5q-,+8,−12	1.5	6
32	64	Male	RUMD	Normal	0.5	1.5
33	67	Female	RUMD	+8,20q-,7q-	1.5	2.5
34	66	Male	RAEB II	−7	3	13
35	63	Male	RAEB II	Normal	2.0	17
36	42	Female	RARS	+8,20q-	0.5	0
37	55	Male	RUMD	7q-	1.5	4.5
38	51	Female	RARS	Normal	0	0
39	56	Male	5q- syndrome	5q-	0.5	1.5
40	64	Female	RA	Normal	0	0.5
41	65	Female	RA	Normal	0	0
42	76	Male	RAEB I	7q-	2	6.5

^a^Cases numbered 8 to 42 were enrolled in our whole experiment while cases numbered 1 to 7 only volunteered in the ELISA and RT-PCR parts.

**Table 2:** Primers and sequence.

Name	Sequence (5′-3′)	Base number
GAPDH	Forward: GCACCGTCAAGGCTGAGAAC	20
	Reverse: TGGTGAAGACGCCAGTGGA	19
IL-17	Forward: CGGCTGGAGAAGATACTGGT	20
	Reverse: TGGTGAAGACGCCAGTGGA	19
ROR*γ*t	Forward: ACTCATCGCCAAAGCATCC	19
	Reverse: AGGTGACTCGGTTTCAGTGC	20
STAT-3	Forward: GAGAAGGACATCAGCGGTAAG	21
	Reverse: CAGTGGAGACACCAGGATATTG	22
